# Carcass characteristics and physicochemical and sensory properties of meat from three species of wild geese hunted in Poland

**DOI:** 10.1016/j.psj.2026.106877

**Published:** 2026-03-28

**Authors:** Paweł Piekarczyk, Piotr Domaradzki, Mariusz Florek, Marek Kowalczyk, Barbara Topyła, Katarzyna Tajchman

**Affiliations:** a Regional Directorate of the State Forests in Lublin, Czechowska 4, Lublin 20-950, Poland; b Department of Quality Assessment and Processing of Animal Products, University of Life Sciences in Lublin, Akademicka 13, Lublin 20-950, Poland; c Department of Animal Ethology and Wildlife Management, University of Life Sciences in Lublin, Akademicka 13, Lublin 20-950, Poland

**Keywords:** Greater White-fronted Goose, Greylag Goose, Bean Goose, Carcass traits, Meat quality

## Abstract

This study aimed to characterize the carcass traits, physicochemical and sensory properties of meat from three wild goose species ‒ Greater White-fronted Goose (*Anser albifrons*), Greylag Goose (*Anser anser*), and Bean Goose (*Anser fabalis*) ‒ and compare them with two domestic breeds, White Kołuda® and Biłgorajska. Forty-five carcasses (nine males of each species/breed) were analysed. Breast and leg muscles were evaluated for proximate composition, pH, colour, water-holding capacity, heme pigments, lipid oxidation (TBARS), and texture, followed by a sensory assessment of cooked breast meat. Statistical comparisons focused on the effect of species (among wild geese) and goose type (wild vs domestic).

Wild geese differed considerably in carcass traits, reflecting inherent interspecific morphometric variation. Among the wild species, Greylag Goose showed the highest body and empty body weights, and edible carcass component weights, while the Greater White-fronted Goose exhibited the lowest values. The White Kołuda® breed (WK), compared with wild geese, displayed the greatest total body weight and heaviest edible parts; however, the percentage of breast muscle was approximately twice as high in wild birds. Domestic goose meat ‒ particularly from WK ‒ contained more intramuscular fat and moisture but less protein, and had lower pH both before and after thawing. In contrast, Biłgorajska geese showed values closer to those of wild birds. Wild geese had darker meat with higher heme pigment content, lower expressible water in breast, and greater susceptibility to oxidation (higher TBARS). Domestic geese, especially Biłgorajska, showed higher shear force and energy in both raw and cooked breast. Sensory evaluation indicated that WK breast had a more intense meat aroma but was less juicy and tender; simultaneously wild geese displayed higher juiciness and tenderness but also a higher intensity of off-odours and off-flavors. Across all groups, overall acceptability exceeded 8 on a 10-point scale. These findings confirm substantial phenotypic and qualitative differences between wild and domestic geese, most likely associated with domestication, including reduced locomotor activity and selection for meat production.

## Introduction

Geese are water birds belonging to the family Anatidae, subfamily Anserinae, mainly assigned to two main genera: *Branta* (‘black’ geese), comprising six species, and *Anser* (‘grey’ and ‘white’ geese), consisting of 11 species (Ottenburghs et al., 2016). The European domestic goose (*Anser anser f. domesticus*) is derived from the Greylag goose (*Anser anser*), and many years of selective breeding have led to numerous genetically varied breeds and genetically distinct lines, which currently have the status of traditional breeds or local varieties in many countries, constituting an important element of the biodiversity of the species (Kozák, 2019). There are currently 14 goose breeds (Biłgorajska, Zatorska, Garbonosa, Kartuska, Kielecka, Lubelska, Podkarpacka, Pomorska, Rypińska, Suwalska, Słowacka, Romańska, Kubańska and Landes) kept in Poland and protected *in situ* in their natural environment under a genetic resources conservation programme. In each protected flock (strain), genetic equilibrium is maintained while preserving the characteristic phenotypic traits of birds of both sexes. Despite this diversity, the dominant position is occupied by the White Kołuda® breed, which provides about 95% of the goose meat produced in Poland (Kokoszyński et al., 2022). This is the W-31 meat crossbred obtained by crossing ♂ W-33 × ♀ W-11, derived from a population of White Italian geese. Since 2012, White Kołuda® has been regarded as a separate breed, often referred to as the Polish national goose. These birds are kept in a semi-intensive system and fattened in a traditional manner with oats to the age of 16–17 weeks, which results in a high-quality product recognized in the European market under the trade name ‘young Polish oat goose’ (Biesiada-Drzazga, 2014; Buzała et al., 2014).

The occurrence of nine species of free-living geese has been recorded in Poland (Checklist of the birds of Poland, 2025); however, hunting law permits the shooting of only three of these – Greylag (*Anser anser*), Bean Goose (*Anser fabalis*) and Greater White-fronted Goose (*Anser albifrons*) (Regulation of the Minister of Environment, 2023). The Egyptian Goose (*Alopochen aegyptiaca*) and Canada Goose (*Branta canadensis*) are also encountered in Poland, but have the status of invasive alien species (Checklist of the Birds of Poland, 2025), which in specified circumstances allows them to be culled (Regulation of the Council of Ministers, 2022).

The Greylag Goose is considered to be the only species that regularly nests in Poland. Its population in Europe is estimated at 797,000‒975,000 individuals (BirdLife International, 2021). The Bean Goose and White-fronted Goose, unlike the Greylag, do not breed in Poland but are present only at their wintering grounds and on migration routes. Their European populations are estimated at 468,000‒647,000 and 1,180,000‒1,970,000, respectively (BirdLife International, 2021).

The current level of annual goose harvesting in Poland (2022/2023 and 2023/2024 seasons) is about 13,000 birds, with a seasonal distribution closely dependent on the course of migration and the population size of each species. In the 2023/24 season, the Bean Goose (48.1%), Greylag Goose (37.7%) and Greater White-fronted Goose (14.2%) made up the largest proportions (Panek and Kolasiński, 2024). As herbivores, wild geese play an important role in ecosystems, but their growing populations cause overgrazing and increase the risk of pathogen transmission and crop damage (Buij et al., 2017), thereby exacerbating the conflict between agriculture and bird conservation (Polakowski et al., 2021; Söderquist et al., 2022). Tools used to limit negative effects caused by geese include hunting and derogation shooting. Carcasses obtained in this manner can be a valuable source of raw meat of high nutritional and culinary value, which, according to the principles of sustainable wildlife management, should be appropriately managed as valuable meat (Geldenhuys et al., 2013; Söderquist et al., 2022). Although game meat remains a niche product in the contemporary food market, due to its limited availability, seasonality, and culling limits, its popularity has been systematically increasing in many countries, which constitutes an additional diversification of the human diet (Florek et al., 2017a). The meat of game animals has always been highly valued by consumers for its nutritional properties and health benefits and is also considered more organic than other types of meat obtained from slaughter animals (Florek et al., 2017a; Tomasevic et al., 2018). Free-living animals feed primarily on natural, varied food, rich in herbs, minerals, and other active substances, which has a beneficial effect on the nutritional and bioactive value of their meat, in addition to imparting distinct organoleptic characteristics (Florek et al., 2017a; Bombik et al., 2022; Quaresma et al., 2024). For this reason, the number of consumers interested in new taste sensations is continually growing. It should be stressed that most of the game consumed is obtained from large game animals (e.g. deer or wild boar), whereas game birds remain little known to a wider group of consumers (Janiszewski et al., 2018; Tomasevic et al., 2018; Söderquist et al., 2022). Therefore, greater focus should be placed on assessment of the meat quality of wild bird species, which may be a valuable alternative to both red and white meat. In addition, this diversification of meat sources may contribute to better adaptation of the market to varied and changing consumer preferences.

The contemporary concept of meat quality, including the meat of wild waterfowl, is broad and multi-faceted. It encompasses not only nutritional value, but also sensory and technological quality and safety (Florek et al., 2017b; Wereńska and Okruszek, 2023). The most commonly determined qualitative properties of poultry meat include proximate composition, ultimate pH, purge loss or thawing loss, cooking loss, water holding capacity (WHC), colour, and sensory properties, which are determined by a number of factors, such as species, sex, diet, and rearing conditions (Wereńska and Okruszek, 2023).

The meat quality of farmed geese has been the subject of numerous studies, especially with regard to commercial crossbreds such as White Kołuda®, Eskildsen Schwer, or Novohradská, as well as native breeds kept in small flocks – in Poland, e.g. Biłgorajska (Adamski et al., 2016; Gumułka and Połtowicz, 2020; Kokoszyński et al., 2022; Lewko et al., 2023; Wereńska and Okruszek, 2023), and in other countries (Tůmová and Uhlířová, 2013; Uhlířová et al., 2018; Boz et al., 2019). In contrast, there is relatively little knowledge of the meat properties of wild geese. Only a few papers are available (Geldenhuys et al., 2013, 2014, 2016a; Söderquist et al., 2022; Abd El Rahman et al., 2024), most of which focus on a single species, i.e. Egyptian Goose (*Alopochen aegyptiacus*) (Geldenhuys et al., 2013, 2014, 2016a; Abd El Rahman et al., 2024). This species, according to current taxonomy, is assigned to the duck subfamily (tribe *Tadornini*), and not to the True Geese (tribe *Anserini*). In consequence, there is a significant knowledge gap concerning variation in the meat quality of European species of wild geese. There is also a lack of comparative studies of the meat quality of geese living in natural conditions vs. farmed geese.

Therefore, the present study aimed to characterize the carcass traits and physicochemical and sensory properties of meat from three species of free-living geese harvested in Poland (Greater White-fronted, Greylag and Bean Goose) and to compare them with those of two selected domestic goose breeds (a commercial meat breed and a conserved breed). In addition, the effect of muscle type (breast vs. leg muscle) was assessed.

## Materials and methods

### Ethical approval

The research was conducted according to the guidelines of the Declaration of Helsinki and in compliance with European Union law (Directive 2010/63/UE, implemented in Poland via Legislative Decree 266/2015) of the European Parliament and of the Council on the protection of animals used for scientific or educational purposes. Ethical review and approval were waived for this study, since according to Polish law, ethical approval is not required for actions within the scope of the Act of 18 December 2003 on animal treatment facilities and agricultural activities, including the rearing or breeding of animals, carried out in accordance with the provisions on the protection of animals. All wild geese were hunted in compliance with the Polish hunting law and collected directly from hunters. The domestic geese were obtained from local farms operating in the small-scale production sector. Farmed birds were slaughtered in accordance with the provisions of Council Regulation (EC) no. 1099/2009 of 24 September 2009 (EC 2009). Furthermore, no experimental procedures were carried out on live animals. The main part of the study was begun after the hunted (wild) or slaughtered (farm) birds had been obtained, and therefore there was no legal basis for requesting approval from the local ethics committee.

### Sample collection and preparation

The materials for the study consisted of 45 goose carcasses, including three free-living species, i.e. Greater White-fronted Goose (GWF; *Anser albifrons, n* = 9), Greylag Goose (GRE; *Anser anser, n* = 9) and Bean Goose (BEA; *Anser fabalis, n* = 9), and two domestic (*Anser anser f. domesticus, n* = 9) breeds: White Kołuda® (WK) and Biłgorajska Goose (BI, *n* = 9).

#### Wild geese

The wild birds were hunter-harvested in the basin of the River Odra in north-western Poland by hunters authorized as members of the Polish Hunting Association, during the hunting season (between December 2022 and January 2023) and in accordance with the pertinent national legislation. The sex of all wild and domestic birds was verified based on gonad examination. Due to the small number of female wild geese, this sex was exluded, and only males and their carcasses were used for the study. Determining the age of individuals in wild goose populations based solely on external appearance is challenging. The exact age of birds can only be determined when individuals have been previously marked (e.g., ringed) at hatching. Without such marking, external morphological traits can generally only be used to distinguish broad age classes, such as goslings, juveniles (immature birds; hatch-year; <1 year), and adults (after hatch-year; >1 year). In the present study, the age of wild geese was therefore estimated based on external morphological characteristics described by Krąkowski (2022) for the studied goose species, including differences in the coloration of the plumage, beak, tarsus, and feet. In general, juvenile geese typically show more uniform grey-brown plumage with weaker colour contrast and lack abdominal markings, whereas adult birds exhibit more distinct plumage patterns and stronger colour contrast, often with visible abdominal spots or bars. Juvenile individuals also display less intense coloration of the legs and a less clearly defined beak coloration. Body mass is generally not considered a reliable criterion for age determination due to its high variability. Importantly, geese hatched in the same year retain visible juvenile morphological traits during the autumn and early winter period. Considering the breeding period of geese (April–June), the time of collection (December–January), and the absence of juvenile morphological characteristics, all wild geese included in this study were classified as older than 18 months.

After harvest the wild birds were placed in plastic bags and transported to the laboratory as quickly as possible (but within 72 hrs post mortem), in insulated containers with cooling inserts to maintain a temperature of about 4 °C throughout the meat. In the laboratory, each bird was weighed to determine its body weight, and the head was removed between the 2nd and 3rd cervical vertebrae. Then the carcass was eviscerated, and the inner organs (heart and liver) were separated from the viscera for determination of empty body weight. For further analyses, breast (IMPS #P4015) and leg (IMPS #P4031) muscles (North American Meat Processors, 2014) were collected from both sides of the carcass, following skinning, trimming of external fat deposits, and also deboning in the case of the legs. Each edible part was weighed, and the percentages in relation to body weight (for offal) and empty body weight (for meat) were calculated.

At 72 h post mortem, pH was measured directly in the breast muscle tissue, whereas torrymeter values were determined in both breast and leg muscles. The breast and leg muscles from each carcass were vacuum-packed separately (MULTIVAC Sepp Haggenmüller SE & Co. KG, Wolfertschwenden, Germany) in PA/PE pouches with a thickness of 90 μm (20 μm PA and 70 μm PE, oxygen permeability of <56 cm^3^/m^2^/24 h/bar at 50% RH, and water vapour permeability of <3 g/m^2^/24 h/bar at 85% RH) and then frozen at −20 °C for 6 months.

An overview of the experimental design and analysis timeline is presented in Figure S1. After thawing for 24 h in a refrigerator at 2 °C (±2 °C), thawing loss was determined. Subsequently, colour and torrymeter values were measured in raw intact breast and leg muscles, while pH was determined in breast muscle. Following these measurements, each breast was divided into two parts. The left half was subjected to heat treatment to determine cooking loss and was then used for sensory evaluation, colour measurement of the cooked meat, and analysis of texture parameters (shear force and MORS), whereas the right half was designated for shear force assessment in the raw state and for grinding. The leg muscle, similarly to the right portion of the breast, was ground and the following analyses were performed in the prepared samples: proximate composition, oxidation–reduction potential (ORP), water activity (aw), expressible water, heme pigments, TBARS, and pH (leg meat only). All physicochemical measurements were performed in at least two replicates for each sample.

#### Domestic geese

Domestic male geese were sourced from two producers in Lublin Voivodeship, Poland. The White Kołuda® geese came from a flock of 1000 individuals, reared and fattened in accordance with the regulations for the ‘Polish young oat goose’. According to this standard, birds are kept in a semi-intensive system until the age of 17 weeks, in open-air runs and at pasture. Up to 14 weeks, the diet is based mainly on green forage and cereal grains, with a small addition of concentrated feeds. From 15 to 17 weeks of age, the geese are fattened on whole grain oats ad libitum. The feed rations were previously described in detail by Wołoszyn et al. (2020). The adult male Biłgorajska geese were selected for the study from a larger flock included in the genetic resources conservation programme. The body weight of the birds ranged from 4.5 to 6.2 kg (average 5.2 kg). Birds were housed in partially shaded open-air runs with an indoor shelter. Geese were provided with prepared pelleted feed twice daily, with ad libitum access to water, and could graze freely during the day in summer. The age of the domestic geese was determined based on farmers’ statements and accompanying documentation. The age of White Kołuda® geese were 17 weeks, whereas Biłgorajska geese were 4 years old (after four reproductive seasons).

Because the Biłgorajska breed is covered by the National Genetic Resources Conservation Programme, the authors only had access to limited research material (muscle tissue), which was insufficient for comprehensive measurements and analyses. Therefore, this study does not include the results of slaughter analysis and sensory evaluation for this breed.

The geese were randomly selected for slaughter and then underwent 12 h of fasting with unlimited access to water. The slaughter procedure was carried out on the farms. They were first stunned, followed by severing of the carotid arteries and immediate bleeding, in accordance with national provisions (Regulation of the Minister of Agriculture and Rural Development, 2015). The carcasses and meat of domestic geese were handled in the same manner as for the wild geese (Figure S1).

### Analyses and measurements

#### Proximate composition

Content of moisture, protein, total fat and total ash were determined according to AOAC proce dures 950.46B, 981.10, 920.39, and 920.153, respectively (AOAC, 1990). The caloric value of 100 g of meat was calculated from the content of total protein and intramuscular fat, using physiological energy values of 4 kcal for protein and 9 kcal for fat (Litwińczuk et al., 2016).

#### pH, oxidation-reduction potential, water activity and torrymeter value

The meat pH was measured using the CP-401 pH-meter (Elmetron, Zabrze, Poland) with automatic buffer detection and temperature compensation. A penetrating glass electrode (ERH-12-6, Hydromet, Gliwice, Poland) was used, calibrated at the two points pH 4.00 and pH 7.00 with high-accuracy pH buffer solutions (±0.02 at 20 °C, Elmetron, Zabrze, Poland). The pH was measured directly in intact breast muscle tissue and in ground leg muscle (Kokoszyński et al., 2022).

Oxidation-reduction potential (ORP) was assessed as described by Nam and Ahn (2002) using the CP-401 digital pH-meter (Elmetron, Zabrze, Poland) set to the millivolt scale (mV), connected to an ERPt-13 platinum combination redox electrode (Hydromet, Gliwice, Poland).

Water activity (a_w_) was measured using a Rotronic HygroLab C1 analyser (Bassersdorf, Switzerland). The AWQ mode with stabilization set to 15 min was applied after conditioning the samples at room temperature (20 °C ± 1 °C) (Kowalczyk et al., 2024). The dielectric properties of muscles were analysed using a torrymeter (GR Torry Fish Freshness Meter, Distell Industries, West Lothian, Scotland), according to the manufacturer’s instructions. The measuring sensors of the device were placed firmly against cleaned and chilled (4–10 °C) muscle tissue, and then the measurement was made. Sensors were cleaned after every measurement (Kowalczyk et al., 2024).

#### Thawing loss, cooking loss and water-holding capacity (WHC)

Thawing loss (TL) from the breast and leg muscles was determined by calculating the difference between the weight prior to freezing (before vacuum-packaging) and the weight of the thawed samples, following gentle blotting with tissue paper before weighing, and expressed as a percentage of the initial weight (Wereńska and Okruszek, 2023).

Cooking loss (CL) was calculated as the percentage difference between the initial weight of the breast sample and that of the sample following heat treatment in a water bath (HENDI GN 1/1 sous-vide system, Rhenen, Netherlands) at 80 °C to an internal temperature of 75°C (in the thickest part of the muscle), after which it was cooled in air to 20–22 °C and wiped with a paper towel to remove visible exudates (Kaliniak-Dziura et al., 2022; Wołoszyn et al., 2020). Then the sensory, colour, and texture traits of the samples were assessed. For this purpose, breast muscle with an average length of 11–15 cm was divided into three parts; the upper part (head, 5–6 cm long) was used for texture analyses, the middle part (the next 3–5 cm) for sensory assessment, and the rest (tail) was discarded.

Expressible water was determined by the filter paper press method (Grau and Hamm, 1953) and expressed in cm^2^. The ring zone of the expressed juice was determined as the difference between the total area and the pressed meat area. The amount of water released is inversely proportional to the water-holding capacity (WHC) of the meat (a higher area of exudative juice indicates a lower WHC) (Demir et al., 2025; Gumułka and Połtowicz, 2020).

#### CIE colour

The colour of the breast and leg muscles was measured using a Konica Minolta CM-600d portable spectrophotometer (Konica Minolta Sensing, Inc., Osaka, Japan) with a pulsed xenon lamp and 8 mm aperture size, including a specular component, illuminant D65, standard observer at 10°, and zero and white calibrations. The results of the measurements were given in the CIE L*a*b* colour space of the Commission Internationale de L’Eclairage (CIE, 2004; Gümüş et al., 2024), including the following spectral values: L* (lightness), a* (redness), and b* (yellowness). The colour values of the surface of raw intact meat samples (on the inner side of the muscles, i.e. adjacent to the bone) were recorded after 30 min of exposure to atmospheric oxygen under PVC wrap at 4 °C to allow blooming (Kokoszyński et al., 2022). To measure the colour of breast muscle after heat treatment, samples from the cooking loss evaluation were used. After the samples had been cooled to room temperature (20–22 °C), the colour was measured on the cross-section (Geldenhuys et al., 2014; Wołoszyn et al., 2020).

#### TBARS and total heme pigments

Lipid oxidation was determined by measuring 2-thiobarbituric acid reactive substances (TBARS) according to Witte, Krause, and Bailey (1970). The absorbance was measured at 530 nm using a Varian Cary 300 Bio spectrophotometer (Varian Australia PTY, Ltd., Mulgrave, Australia) and expressed in mg of malondialdehyde (MDA) per kg of sample.

Total content of heme pigments was determined according to Hornsey’s (1956) method using an acetone/HCl mixture. The absorbance was measured using a Varian Cary 300 Bio spectrophotometer (Varian Australia PTY, Ltd.) at 640 nm against a blank sample and expressed in μg of hematin per g of meat.

#### Meat texture

The MORS force and energy of cooked breast were measured with a TA.XT plusC Texture Analyser (Stable Micro Systems, Godalming, UK) equipped with a 5-kg load cell and a Meullenet-Owens Razor Shear Blade (A/MORS, Stable Micro Systems). The test was conducted at the following device settings: pretest speed 2 mm/s, test speed 10 mm/s, trigger force 10 g, and penetration depth 15 mm. Each breast was stabbed five times perpendicular to the muscle fibres, keeping a distance of 15–20 mm from each measurement point. After 100 insertions, the MORS blade was replaced to ensure sharpness. MORS shear force is reported as the maximum shear force (N) and MORS shear energy as the area under the force–deformation curve (N × mm) (Lee et al., 2008). Shear force (N) and shear energy (N × mm) were measured on both raw and heat-treated breast muscle using the Zwick/Roell Proline BDO125 FB0.5TS universal testing machine (Zwick GmbH & Co, Ulm, Germany) with a V-shaped shear blade and crosshead equipped with a 500 N load cell. Strips of muscle (10 × 10 mm, about 20 mm long) with a cross-sectional area of 1 cm^2^ were cut parallel to the longitudinal orientation of the muscle fibres and sheared at room temperature (20–22 °C) perpendicular to the fibres, at a crosshead speed of 100 mm/min (Geldenhuys et al., 2014; Gronowicz et al., 2018).

#### Sensory evaluation

The sensory properties of the breast muscle from four genetic groups (GWF, GRE, BEA, and WK) were evaluated on warm samples (50 °C), after cooking loss determination, by six non-smokers of both sexes (30 to 45 years of age) with experience in meat sensory analysis. Prior to testing, the panellists took part in a preparatory session regarding the descriptive profile of the sensory attributes, to allow them to discuss and clarify each attribute to be evaluated. Samples were cut into rectangles approximately 2 cm long and 1.5 cm wide, coded with three-digit numbers, and served to the panellists. All testing was carried out under controlled conditions (room temperature of approx. 20 °C, free of noise and odour, under fluorescent lighting). Water and fresh bread were provided to clean and refresh the palate between samples. The sensory analysis was carried out using the scaling method (PN-ISO 4121 1998). A 10 cm long unstructured linear graphical scale with a specific edge point was used to indicate the assessment of the following attributes: meat aroma (undetectable; very intense), game aroma (undetectable; very intense), off-odours (undetectable; very intense), juiciness (very dry; very juicy), tenderness (very tough; very tender), meat flavour (undetectable; very intense), game flavour (undetectable; very intense), off-flavours (undetectable; very intense) and overall quality (highly undesirable; extremely desirable). The sensory evaluation protocol was based on the methodology described by Kaliniak-Dziura et al. (2022) with sensory descriptors selected according to recommendations for wild bird meat reported by Geldenhuys et al. (2014) and Söderquist et al. (2022), considering the characteristics of the samples analysed in this study.

### Statistical analysis

The data were subjected to statistical analysis by STATISTICA v. 13 (TIBCO Software Inc., Palo Alto, CA, USA). The analysis was limited to a one-way analysis of variance testing the influence of goose species (as a main effect) on carcass and meat quality among wild birds, and differences were compared using Tukey’s (HSD) test. The means for goose domestic breeds (WK vs. BI) and goose type (wild vs. domestic) were compared by Student’s t-test. The same statistical analysis was used for muscle comparison (breast vs. leg) within the species/breed or goose type for the proximate composition and intrinsic properties. The sensory properties of cooked meat were analysed using the Nonparametric Statistics package. The influence of goose species was tested by the Kruskal–Wallis test (comparison of many independent groups), and the impact of goose type was determined by the Kolmogorov–Smirnov test (comparison of two independent groups). The results are given in tables as the mean value for each dependent variable and the standard error of the mean.

## Results and discussion

### Carcass characteristics

Basic carcass traits and most edible carcass components were significantly (*P* < 0.05) dependent on the species of wild goose and the type of bird (Table 1). The highest body weight, empty body weight, and head and viscera weight were obtained for Greylag Goose (GRE) and were significantly (*P* < 0.05) different from the values for Greater White-fronted Goose (GWF), and in the case of viscera weight for Bean Goose (BEA) as well. The slaughter characteristics of wild geese have very seldom been the subject of research. The size of individual goose species is highly varied, and their body weight and carcass composition depend on numerous biological and environmental factors, the most important of which include species, age, sex, physiological state (moulting period, breeding period, or migratory behaviour) and habitat, particularly food availability and quality (Bromley and Jarvis, 1993; Fox and Kahlert, 2005; Geldenhuys et al., 2013). The smallest species are the Black-bellied Brent (*Branta bernicla nigricans*) and Barnacle Goose (*Branta leucopsis*), with weight ranging from 0.88 to 1.79 kg and from 1.21 to 2.40 kg, respectively, while the remaining species are medium-sized or large birds (Dunning, 2007; del Hoyo et al., 2016). The body weight of GWF geese has been shown to range from 2.05 to 3.6 kg, and that of BEA from 2.69 to 4.06 kg, with males usually weighing more than females (Dunning, 2007; del Hoyo et al., 2016). The Greylag Goose, which is the protoplast of the European domestic goose, is considered one of the largest of the so-called grey geese of the genus *Anser*, with body weight ranging from 2.50 to 4.56 kg (Dunning, 2007; Kozák, 2019). The live weight range of 1714.7–2614.0 g reported for male wild Egyptian geese (*Alopochen aegyptiacus*) (Geldenhuys et al., 2013; Abd El Rahman et al., 2024) is lower than the values obtained for birds in the present study. Similarly, the head weight of Egyptian geese amounting to 64.9 g (average for males and females) was lower, but its percentage (4.25%) of the total live body weight (Abd El Rahman et al., 2024) was similar to that obtained in the present study (on average 4.17%).

**Table 1:** Carcass traits, weights, and proportions of edible carcass components in wild and domestic geese (means; SEM).

Item	Wild			**Average**	SEM	Domestic	SEM
	Greater White-fronted (GWF)	Greylag (GRE)	Bean (BEA)			White Kołuda (WK)	
Body weight^1^, g	2346.7a	3471.1b	2915.2ab	**2863.6x**	130.3	6693.2y	182.3
Empty body weight^2^, g	1792.7a	2721.1b	2411.9b	**2257.0x**	110.0	5389.0y	158.5
Head, g	97.0a	146.5b	120.3ab	**119.3x**	5.5	284.9y	9.7
Viscera weight^3^, g	395.4a	538.3b	328.9a	**426.4x**	25.2	831.5y	23.9
*Edible components*							
Liver, g	43.7a	65.5b	39.0a	**49.8x**	3.5	116.1y	5.8
Liver, %	1.9b	1.9b	1.3a	**1.8**	0.1	1.7	0.1
Heart, g	20.7a	35.1b	24.9a	**26.5x**	1.7	41.7y	2.8
Heart, %	0.9	1.0	0.8	**0.9y**	0.0	0.6x	0.0
Breast meat^4^, g	424.7a	622.5b	489.9a	**506.9**	21.2	569.3	17.3
Breast meat, %	23.9	23.4	20.4	**22.9y**	0.6	10.6x	0.5
Leg meat^4^, g	124.2a	234.9b	148.3a	**167.2x**	11.9	560.1y	38.5
Leg meat, %	6.9a	8.9b	6.2a	**7.4x**	0.3	10.4y	0.7

a, b - means with different letters in a row are significantly different at *P* < 0.05 (effect of goose species); x, y - means with different letters in a row are significantly different at *P* < 0.05 (effect of goose type – wild vs. domestic White Kołuda®); SEM – standard error of the mean.

1 Body weight of the goose after shooting or after bleeding for domestic geese;

2 Weight of the goose carcass after evisceration with feathers and feet;

3 Edible and inedible internal organs;

4 Weight of meat without skin and bones.

The variation in body mass and organ weight observed among the wild geese in this study primarily reflects inherent morphometric differences among species of the genus *Anser*, with larger species naturally exhibiting greater body and organ weights. In addition, species-specific ecological strategies during the wintering period may further influence these traits by affecting energy intake and expenditure. Previous studies have shown that during autumn and winter GRE geese frequently forage on arable land, particularly on harvested fields and croplands that provide high-energy food resources (de Jager et al., 2026; Olsson et al. 2025). In contrast, GWF often use a broader range of habitats, including both agricultural fields and natural grasslands, feeding on cereals and sedges (Ely and Raveling, 2011; Fox et al., 2017). Differences in habitat use and diet composition may therefore influence the availability of metabolizable energy and nutrient intake, which in turn can affect body condition and carcass traits. Moreover, wintering individuals allocate a considerable proportion of daily activity to foraging and movement between feeding sites, which may further increase energy expenditure (VonBank et al., 2021).

The carcass parameters obtained for the domestic White Kołuda® goose (WK) were 2–3 times as high as the average values in the group of wild geese (*P* < 0.01; Table 1). Domestic geese were originally similar in size to their wild ancestors, and while geese are believed to have changed less during domestication than other livestock animals, selection has made them larger and heavier, thereby reducing their ability to fly. The goose is one of the fastest-growing species of domestic poultry. Birds living in the wild develop only to the weight limit characteristic of the species, which is restrained by their nutrient uptake capacity. Rapid growth and early fattening are modern physiological traits that have resulted from domestication (Kozák, 2019). Meat breeds of geese are ready for sale at the age of just 10 weeks, and depending on the type of feeding, reach a live weight from 4.5 to 5.5 kg at the age of 12–15 weeks (Kozák, 2019). The results obtained for WK correspond to literature reports indicating that geese attain a body weight from 5.8 to 8.3 kg in the final fattening period (15–17 weeks) and exhibit very good productive and slaughter parameters (Lewko et al., 2023).

### Edible carcass components

As in the case of carcass traits, the weights of the edible parts of the carcass (liver, heart, breast meat, and leg meat) among wild geese were significantly the highest for GRE (GRE vs. GWF and BEA; *P* < 0.05; Table 1). Detailed literature data for weight and proportions of edible organs in wild geese are very scarce. For Egyptian Goose, Abd El Rahman et al. (2024) report lower weights and percentages for the liver and heart, 19.7 g (1.29%) and 17.6 g (1.15%), respectively, than those obtained in the present study. The weight of the most valuable element of wild goose carcasses, i.e. the breast, ranged from 394.3 g (Abd El Rahman et al., 2024) to 491.8 g (Geldenhuys et al., 2013) in male Egyptian geese, which is comparable to the breast weight of GWF (424.7 g) and BEA (489.9 g) but lower than in GRE (622.5 g) (Table 1). Similarly, Söderquist et al. (2022) indicate significant variation in breast muscle weight between four wild goose species (Barnacle Goose < BEA < GRE < Canada Goose), although the range reported by the authors (130.4 g ‒ 308.2 g) is considerably lower than that obtained in the present study. The leg muscle weight in the wild goose species evaluated in this study (124.2–234.9 g) was lower than for Egyptian geese (265.7–280.1 g; Geldenhuys et al., 2013; Abd El Rahman et al., 2024). The higher percentage of edible carcass elements in Egyptian geese (29.5–34.8% for breast and 18.8–21.0% for leg (thigh + drumstick)) compared to the present study (20.4–23.9% for breast and 6.2–6.9% for leg; Table 1) was most likely linked, apart from species differences, to the fundamentally different method of estimating them. Abd El Rahman et al. (2024) and Geldenhuys et al. (2013) calculated them in relation to the eviscerated carcass without feet, feathers, skin, head, or neck, whereas in the present study they were calculated in relation to empty body weight (the whole carcass except the head and viscera).

Comparison of the average edible carcass components of the three wild goose species with the domestic goose representative showed that, while the WK breed had significantly (*P* < 0.05) higher liver, heart, leg meat weight and numerically higher breast meat mass (*P* > 0.05), the relative proportions (%) of heart and breast meat were significantly greater in the wild geese (*P* < 0.05; Table 1), most likely due to their greater activity. Domestication of geese has been shown to have shortened organs involved in metabolism (e.g. the duodenum, jejunum, and ileum), while enlarging the liver (Kozák, 2019). Currently, liver weight in domestic goose breeds can reach 175 g, and under specific handling and fattening conditions inducing spontaneous hepatic steatosis (without force-feeding), it can even reach up to 500 g (Fernandez et al., 2016). In non-overfed domestic geese, however, liver weight typically ranges from 64 to 124 g and heart weight from 25.5 g to 47.8 g, accounting for 1.4–3.4% and 0.55–1.19% of body weight, respectively (Murawska, 2013; Aslan and Öztürk, 2022; Aslan et al., 2024; Wegner et al., 2025). These patterns are confirmed by the results of the present study (Table 1).

The most valuable parts of a goose carcass are the breast and leg, which as culinary parts are the most commonly eaten by consumers. The muscles of wild geese are adapted to flight and endurance during migration, and at the same time they weigh less than in domestic breeds, especially those selected for meat purposes. Moreover, domestic geese walk more and fly less, and due to their higher body weight, their leg bones are thicker, while the wing bones are shorter and wider than in wild geese (Mannermaa, 2018; Kozák, 2019). Despite these changes, leg musculature has changed relatively little, whereas the muscles of the wings and breast have decreased by about 30% (Kozák, 2019). Although the weight of the leg meat of the domestic goose WK in the present study was about three times that recorded in wild geese (which does not fully reflect the differences described above), it should be noted that the statistical analysis did not confirm a significant difference (*P* > 0.05) between mean breast weights in the two types of birds (506.9 g vs. 569.3 g). On the other hand, the percentage of breast in wild geese was more than two times that noted in White Kołuda® (22.9% vs. 10.6%; *P* < 0.05), while leg meat accounted for 7.4% of empty body weight in wild geese and 10.4% in domestic geese (*P* < 0.05) (Table 1). In domestic geese, breast muscle typically accounts for 12.1% to 21.9% of carcass weight, and thigh meat for between 9.5% and 17.0%, which can be influenced by diet, breed, and age at slaughter (Adamski et al., 2016; Uhlířová et al., 2018; Boz et al., 2019; Gumułka and Połtowicz, 2020). However, local breeds of geese exhibit lower muscle weight but a more favourable carcass muscle percentage than commercial hybrids such as White Kołuda® (Lewko et al., 2023). It should also be noted that, due to the lack of intense selection, native breeds display greater genetic diversity and are therefore closer to their wild ancestor, the Greylag Goose. Murawska (2013) and Uhlířová et al. (2018) also reported that breast muscle percentage increased with age, whereas thigh percentage decreased, indicating an age-related redistribution of muscle from the leg to the breast.

### Proximate composition

Among the wild goose species, a number of significant differences were noted in the content of the basic chemical constituents of the meat. In the breast muscle, moisture was significantly the lowest in GRE and significantly the highest in GWF (*P* < 0.05). The protein content in GRE and BEA was significantly higher than in GWF (*P* < 0.05), and the fat and ash contents in the breast muscle of all three wild goose species were similar, averaging 2.84% and 1.12%, respectively (*P* > 0.05) (Table 2). Given the similar level of intramuscular fat, the higher energy value of the breast meat was due to the higher protein content in GRE compared to GWF (*P* < 0.05), whereas the higher caloric value of the leg meat was determined by the amount of fat (BEA vs. GRE and GHF, *P* < 0.05). The meat of wild geese offers a number of nutritional benefits making it a valuable addition to a balanced diet. In contrast to our research, Söderquist et al. (2022) showed no significant differences in the proximate composition of breast meat from four species of wild geese, including two species of the genus *Anser* (GRE and BEA) and two of the genus *Branta* (Canada and Barnacle goose). However, the average water content (70.6%), protein content (24.1%), and energy value of 100 g of meat (128.8 kcal) were similar to the results of the present study. The fat content in the meat of GRE, BEA and Barnacle Goose ranged from 2.58% to 2.90%, which is also consistent with our observations. The intramuscular fat amount in Canada Goose, on the other hand, was markedly higher (4.17%), although the differences were not significant.

**Table 2:** Proximate composition and energy value of meat from three species of wild geese and two breeds of domestic geese (means; SEM).

Item	Goose type	Wild			**Wild** **Average**	SEM	Domestic		**Domestic** **Average**	SEM
	Muscle	Greater White-fronted (GWF)	Greylag (GRE)	Bean (BEA)			White Kołuda (WK)	Biłgorajska (BI)		
Moisture										
	Breast	71.19b	70.30a	†71.03ab	**†70.81x**	0.15	†71.94	72.28	**†72.08y**	0.08
	Leg	70.51b	69.75ab	†68.91a	**†69.79x**	0.21	†70.33a	71.94b	**†70.98y**	0.22
Protein										
	Breast	†24.58a	†26.11b	†25.92b	**†25.50**	0.19	†24.33a	†27.09b	**†25.43**	0.35
	Leg	†22.91	†23.25	†22.65	**†22.97**	0.12	†21.14a	†24.50b	**†22.48**	0.40
Fat										
	Breast	†2.83	†2.85	†2.83	**†2.84x**	0.07	†3.91b	†2.81a	**†3.47y**	0.14
	Leg	†6.08a	†6.69a	†8.55b	**†6.98**	0.26	†9.02b	†5.47a	**†7.60**	0.44
Ash										
	Breast	1.14	1.15	1.04	**1.12x**	0.02	†1.26	1.29	**†1.27y**	0.01
	Leg	1.15	1.09	1.06	**1.10x**	0.02	†1.12	1.24	**†1.17y**	0.02
Energy value (kcal/100 g)										
	Breast	†123.8a	†130.1b	†129.1ab	**†127.5x**	1.0	†132.5	†133.6	**†133.0y**	0.9
	Leg	†146.3a	†153.2a	†167.6b	**†154.7**	2.2	†165.7b	†147.2a	**†158.3**	2.7

a, b - means with different letters in a row are significantly different at *P* < 0.05 (effect of goose species or breed - significance for three species of wild geese or two domestic breeds); x, y - means with different letters in a row are significantly different at *P* < 0.05 (effect of goose type – wild vs. domestic); † - means denoted with symbol in a column are significantly different at *P* < 0.05 (effect of muscle type – breast vs. leg); SEM – standard error of the mean.

In the leg muscle (as for the breast muscle), water content was highest in GWF and lowest in BEA (*P* < 0.05). The latter breed had significantly the most intramuscular fat in the leg (8.55%; BEA vs. GWF and GRE, *P* < 0.05), while no significant differences between species were shown for protein or ash (Table 2).

Comparison of wild and domestic geese revealed significantly higher water content in the latter (*P* < 0.05), in both the breast muscle (72.08%) and the legs (70.98%), in comparison to wild geese (70.81% and 69.79%, respectively; Table 2). Significant differences were also shown for the fat content and energy value of the breast meat and for the content of ash in both muscles, i.e. higher levels of these components in domestic geese than in wild geese. Protein content, on the other hand, was similar in the muscles of both types of geese. It should be noted that the two breeds of domestic geese also differed significantly in the content of protein and fat in the breast muscle and of water, protein, fat, and energy in the leg. More water and protein were found in BI geese, and more fat and calories in WK. The water content of breast and leg muscles of domestic geese usually ranges from 70.3% to 75.5% and from 68.8 to 74.9%, while protein content is 20.3–23.3% and 20.0–21.4%, and fat content is 2.4–7.0% and 2.7–10.5% (Haraf, 2014; Munekata et al., 2019). The proximate composition of goose meat may be influenced by factors such as the breeding system, feeding intensity, genotype, or sex (Uhlířová et al., 2018; Boz et al., 2019; Kokoszyński et al., 2022). The results of the present study confirm earlier observations (Puchajda-Skowrońska et al., 2006; Uhlířová et al., 2018) that commercial geese (like the WK breed in the present study) usually contain more fat than native breeds (like the BI breed in the present study). This is because commercial geese are reared for intensive meat production, so they grow faster and attain the inflection point faster than local breeds.

Regarding the differences between muscles, the leg muscles of all geese evaluated in the experiment had lower protein content (on average by 2.74%; *P* < 0.05) and higher fat content (on average by 4.13%; *P* < 0.05) than the breast muscles, which also resulted in higher energy content (on average by 26.2 kcal/100 g; *P* < 0.05). The leg muscle also contained less water (on average by 1.06%; *P* < 0.05), particularly in the wild goose BEA and domestic goose WK (Table 2). Leg muscle typically has higher fat content and lower content of protein and water than the breast (Puchajda-Skowrońska et al., 2006; Łukaszewicz et al., 2016; Gumułka and Połtowicz, 2020; Kokoszyński et al., 2022; Razmaitė et al., 2022). In addition, Geldenhuys et al. (2013) demonstrated a negative relationship between fat content and moisture content in the meat of Egyptian Goose.

### Meat pH

The pH of the breast muscle of wild geese at 72 h after slaughter was similar (on average 5.76) and significantly (*P* < 0.05) higher than in domestic geese (5.69; Table 3); in the latter group, however, a significant difference in pH was noted between the WK and BI breeds (5.63 vs. 5.76, *P* < 0.05). The pH of goose meat is a key indicator of its quality, affecting tenderness, colour, and water-holding capacity. In most studies, routine measurement of goose meat pH has been performed at 24 h post mortem, but Kokoszyński et al. (2022) point out that no standards have been established. Breast muscle pH is found in a wide range from 5.65 to 6.20 (Wereńska and Okruszek, 2023). The literature is also not in agreement as to the effect of the genetic origin of geese on the pH value. Boz et al. (2019), Okruszek et al. (2008), and Tůmová and Uhlířová (2013) found a significant effect, whereas Kokoszyński et al. (2022), Uhlířová et al. (2018) and Gumułka and Połtowicz (2020) observed only non-significant differences in pH between genotypes.

**Table 3:** Meat pH, torrymeter value, oxidation-reduction potential (ORP) and water activity (a_w_) of breast and leg muscles from three species of wild geese and two breeds of domestic geese (means; SEM).

Item	Goose type	Wild			**Wild Average**	SEM	Domestic		**Domestic Average**	SEM
	Muscle	Greater White-fronted (GWF)	Greylag (GRE)	Bean (BEA)			White Kołuda (WK)	Biłgorajska (BI)		
pH_72h_										
	Breast	5.77	5.75	5.74	**5.76x**	0.01	5.63a	5.76b	**5.69y**	0.02
pH_after freezing_										
	Breast	†5.88	†5.84	†5.85	**†5.86x**	0.01	†5.74a	†5.87b	**†5.79y**	0.01
	Leg	†6.24b	†6.04a	†6.30b	**†6.17**	0.02	†6.09	†6.18	**†6.14**	0.01
Torrymeter value_72h_										
	Breast	†3.88	†3.96	†3.70	**†3.86x**	0.05	†6.23	†7.01	**†6.46y**	0.27
	Leg	†5.56	†5.84	†5.35	**†5.59x**	0.11	†13.62b	†10.81a	**†12.69y**	0.27
Torrymeter value_after freezing_										
	Breast	†3.39b	†3.04a	†3.39b	**†3.27x**	0.04	†2.80a	†5.06b	**†3.66y**	0.09
	Leg	†4.95b	†4.02a	†4.77b	**†4.60y**	0.06	†3.73a	†4.33b	**†4.09x**	0.09
ORP_after freezing_										
	Breast	†154.3	†132.0	148.4	**†145.3**	5.6	†126.7	137.4	**†131.1**	4.7
	Leg	†251.3ab	†243.8a	267.0b	**†253.6x**	2.8	†189.0	186.0	**†187.5y**	2.8
a_w after freezing_										
	Breast	0.973c	†0.964b	†0.946a	**†0.960x**	0.002	0.973	0.973	**0.973y**	0.002
	Leg	0.972	†0.970	†0.973	**†0.972**	0.001	0.973	0.974	**0.974**	0.002

a, b, c - means with different letters in a row are significantly different at *P* < 0.05 (effect of goose species or breed - significance for three species of wild geese or two domestic breeds); x, y - means with different letters in a row are significantly different at *P* < 0.05 (effect of goose type – wild vs. domestic); † - means denoted with symbol in a column are significantly different at *P* < 0.05 (effect of muscle type – breast vs. leg); SEM – standard error of the mean.

After thawing, the pH of the breast muscle in wild geese showed similar patterns as for the earlier measurement (72 h post mortem) (Table 3). The pH range in the breast of wild geese ranged from 5.84 to 5.88, and the mean value (5.86) differed significantly from that obtained for domestic geese (5.79), with no significant difference shown between the WK and BI breeds (5.74 vs. 5.87, *P* < 0.05). The pH of the thawed leg muscles in all genetic groups of geese was above 6.0 (leg muscle vs. breast muscle; *P* < 0.05). Among wild geese, pH was significantly (*P* < 0.05) the lowest in the legs of GRE geese (6.04), compared to GWF (6.24) and BEA (6.30). No significant differences were found for this parameter between domestic breeds. An increase in the pH of poultry meat (including goose meat) during frozen storage is fairly well documented in the literature, and the main reason for this phenomenon is identified as the release of proteolysis products of proteins, especially amino acids such as histidine (Wereńska and Okruszek, 2023). In addition, the present study confirms earlier observations by other authors indicating that pH is higher in thigh muscle than in breast muscle in commercial hybrids (Tůmová and Uhlířová, 2013), local breeds of geese (Boz et al., 2019; Razmaitė et al., 2022; Haraf et al., 2023), and wild Egyptian geese (Geldenhuys et al., 2013), which is linked to differences in the composition of muscle fibre types (Geldenhuys et al., 2013; Boz et al., 2019). This difference may also be related to the fact that glycogen is less abundant in leg than in breast muscle, resulting in less intensive post-mortem glycolysis and consequently lower lactic acid production and a higher ultimate pH (Okruszek et al., 2012).

### Torrymeter and oxidation-reduction potential (ORP) values

Torrymeter value can be an indicator of meat freshness, with higher values typically reflecting better quality (Sujiwo et al., 2018). The torrymeter value at 72 h post mortem in the group of wild geese was similar and remained low in both the breast muscle (3.70–3.96) and the leg muscle (5.35–5.84). The means for this parameter in wild geese (3.86 and 5.59) were significantly (*P* < 0.05) lower than for domestic geese – 6.46 in the breast and 12.69 in the legs (Table 3). The significantly lower torrymeter value of the meat of wild geese in comparison with domestic geese was most likely linked to the way the birds were obtained (hunting vs. slaughter on the farm), as the site of dissection (Bae et al., 2014) and handling conditions (Sung et al., 2013) have been shown to significantly affect this parameter in poultry meat. These differences also seem to confirm the inverse correlation between pH and torrymeter readings reported for poultry meat (Sujiwo et al., 2018). In the present study, leg muscles and wild geese exhibited higher pH values compared with breast muscles and domestic birds, which corresponded to lower torrymeter values.

Torrymeter value has also been shown to reliably distinguish fresh and frozen-thawed poultry meat, with lower values obtained for meat kept in frozen storage (Sung et al., 2013; Bae et al., 2014). Freezing leads to the formation of ice crystals that damage cell membranes, leading to efflux of intracellular ions and influx of extracellular ones, which alters the conductivity and permittivity of the tissue (Bae et al., 2014). As a result, torrymeter values in frozen-thawed muscles are generally lower. The decline in torrymeter value during cold storage in the present study confirms earlier observations by other authors (Sung et al., 2013; Bae et al., 2014; Sujiwo et al., 2018). The results obtained at 72 h post mortem (Table 3) were similar to those reported for duck breast (Sung et al., 2013) and chicken thigh (Bae et al., 2014) and at the same time lower than for chicken breast (Sujiwo et al., 2018). After thawing there was a minor numerical decrease in torrymeter values in wild geese (on average by 0.59 units for breast and 0.99 units for leg), while the decrease in domestic geese was considerable (on average by 2.80 units for breast and 8.60 units for leg). This was particularly evident in the WK breed, whose muscles had significantly (*P* < 0.05) lower torrymeter values (2.80 for breast and 3.73 for leg) than BI (5.06 and 4.33, respectively). Similar significant (*P* < 0.05) differences were found in the group of wild geese in the case of GRE (3.04 for breast and 4.02 for leg) in comparison to BEA and GWF (3.39 for breast and 4.77–4.95 for leg) (Table 3).

The oxidation-reduction potential of the breast muscle did not differ significantly between wild species (132.0–154.3 mV), domestic breeds (127.7–137.4 mV), or types of geese (145.3 vs. 131.1 mV). However, significant differences were shown for this parameter in the leg muscles between species of wild geese and between types of geese (Table 3). In addition, the leg muscle had significantly higher ORP than the breast muscle (on average by about 108.3 mV in wild geese and 56.4 mV in domestic geese), and significantly (*P* < 0.05) higher values (on average by 66.1 mV) were noted in the group of wild geese than in in domestic geese.

### Water activity

Water activity (a_w_) in the breast muscle of wild geese varied significantly (*P* < 0.05) from 0.946 to 0.973, differentiating species in the following order: BEA > GRE > GWF. The average a_w_ value for this group (0.960) was significantly lower (*P* < 0.05) than in domestic geese (on average 0.973). In the leg muscle, no significant differences in a_w_ were shown between species, breeds, or types of geese. In the group of wild geese, the average value in the legs (0.972) was significantly (*P* < 0.05) higher than in the breast muscle (0.960). Water activity is a crucial parameter for assessing the shelf-life of food, reflecting water availability essential for the growth of microbes and for chemical and biochemical reactions during storage. The values observed in the present study are typical for fresh poultry meat (Srikanthithasan et al., 2025) which has not undergone processes reducing the content or availability of water (Kowalska et al., 2023). At the same time, irrespective of the differences shown, the range of values obtained indicates that the meat samples were highly susceptible to microbiological changes. Most microbes responsible for unfavourable changes and the decomposition of meat, as well as most pathogenic microbes, require a_w_ values from 0.95 to 0.99; however, values below 0.6 are essential to ensure the microbiological stability of food during storage (ICMSF, 2001).

### Thawing loss, cooking loss and water-holding capacity

Thawing loss (TL) from the meat of wild and domestic geese was similar (*P* > 0.05), averaging 5.3% and 5.9% in the breast muscle and 2.6% and 2.3% in the leg muscle, respectively (Table 4). Irrespective of the species, breed, and type of geese, thawing loss was significantly (*P* < 0.05) lower for the legs than for the breast muscle. Due to the seasonal nature of harvesting of wild geese (the hunting season) and farm geese and the relatively short shelf-life of the meat, methods are needed to preserve goose meat and ensure that it is available year-round. Freezing is considered one of the most important strategies for long-term preservation of the quality of fresh meat. Thawing loss is regarded as one of the most important indicators of the quality of frozen meat and depends mainly on the degree of damage to the histological structure of the muscle tissue and denaturation of proteins. In comparison to the results of the present study, lower TL values for the meat of WK geese were reported by Wereńska and Okruszek (2023). As frozen storage time increased, the authors observed significantly greater weight loss, amounting to 1.36% for breast muscle and 1.21% for leg muscle after six months. These differences with respect to the present study may be due to different ways of preparing the samples, as the muscles in the study cited were frozen together with the skin and subcutaneous fat, whereas the muscles alone, without these tissues, were stored in our study. On the other hand, our results (Table 4) are similar to those reported by Maiboroda et al. (2024), who after three months of frozen storage obtained TL ranging from 3.05% to 3.28% for drumsticks of geese of the Legart Danish breed.

**Table 4:** Water holding capacity indices of meat from three species of wild geese and two breeds of domestic geese (means; SEM).

Item	Goose type	Wild			**Wild** **Average**	SEM	Domestic		**Domestic** **Average**	SEM
	Muscle	Greater White-fronted (GWF)	Greylag (GRE)	Bean (BEA)			White Kołuda (WK)	Biłgorajska (BI)		
Thawing loss, %										
	Breast	†5.3	†5.1	†5.4	**†5.3**	0.3	†6.8	†5.1	**†5.9**	0.4
	Leg	†2.2	†2.9	†2.7	**†2.6**	0.2	†2.5	†2.1	**†2.3**	0.2
Expressible water, cm^2^										
	Breast	†0.7	†0.2	†0.3	**†0.4x**	0.1	3.5	3.0	**3.3y**	0.2
	Leg	†2.9a	†4.3b	†2.8a	**†3.4**	0.2	3.8	3.4	**3.6**	0.2
Cooking loss, %										
	Breast	19.6	20.4	20.7	**20.2**	0.5	26.7b	17.6a	**22.4**	1.2
Time of cooking, min.										
	Breast	29.3	31.9	32.6	**31.3**	0.9	29.5	32.9	**31.1**	0.6

a, b - means with different letters in a row are significantly different at *P* < 0.05 (effect of goose species or breed - significance for three species of wild geese or two domestic breeds); x, y - means with different letters in a row are significantly different at *P* < 0.05 (effect of goose type – wild vs. domestic); † - means denoted with symbol in a column are significantly different at *P* < 0.05 (effect of muscle type – breast vs. leg); SEM – standard error of the mean.

As a result of the freezing-thawing process, the structure of meat becomes looser, leading to a decrease in water-holding capacity and in consequence often to loss of meat juices during cooking as well (Wereńska and Okruszek, 2023). One of the most important properties influencing the quality and economic value of meat is its water-holding capacity (WHC), which affects its juiciness and tenderness, as well as changes in water content during transport, storage, and cooking (Irie et al., 1996). The most important methods for measuring WHC include the filter paper press method (enabling measurement of the amount of expressible water, i.e. loosely bound-water, from meat) and determination of cooking loss. Expressible water from the breast muscle of wild geese took on very low values (0.2–0.7 cm²), which significantly (*P* < 0.05) distinguished it from the breast of domestic geese (3.0–3.5 cm²), as well as from the leg muscles (average 3.4 cm^2^; Table 4). In the leg muscles, this property did not depend on the type of geese (wild vs. domestic *P* > 0.05), but it should be noted that it varied significantly (*P* < 0.05) within the group of wild geese, with the highest value observed for GRE (4.3 cm^2^), in comparison to BEA and GWF (2.8 and 2.9 cm^2^, respectively). Haraf et al. (2023) found that the magnitude of the loss measured by the filter paper press method was significantly influenced by the breed of Polish native geese (Kartuska, Suwalska, Lubelska or Kielecka) and the muscle (breast vs. thigh muscle). In another study, Weng et al. (2021) showed that the breast and thigh muscles of older geese (90 and 120 days) had better WHC (less expressible water) than geese at the age of 70 days. This observation may partially explain the results of the present study, as the wild geese were above the age of 18 months, whereas the WK geese were 17 weeks old. Furthermore, the breast muscle of WK had significantly the lowest pH, which directly influences WHC. As pH decreases towards the isoelectric point of myofibrillar proteins, their net charge is reduced, thereby limiting their ability to bind water and increasing the amount of expressible water, which may partly explain the higher water losses observed in this group (Huff-Lonergan and Lonergan, 2005). In addition, recent proteomic studies have shown that the WHC of the breast muscle of geese is closely linked to the level and type of muscle proteins, especially structural proteins, metabolic enzymes, antioxidant enzymes, and stress response proteins (Zhang et al., 2019). Therefore, there is a need for further studies on the proteome profiles of wild goose meat, which could provide deeper insight and explain very high water-holding capacity.

Cooking loss (CL) in the breast muscle of wild geese was very similar (19.6–20.7%) and averaged 20.2%, and at the same time it did not differ significantly from the group of domestic geese (average 22.4%); however, the BI breed (17.6%) differed significantly (*P* < 0.05) from WK (26.7%) (Table 4). Geldenhuys et al. (2016a) reported higher CL values (28.80–30.31%), dependent on sex and season, for the breast of wild Egyptian geese following heat treatment (internal temp. 71 °C). Söderquist et al. (2022) demonstrated a significant effect of the species of wild geese on the CL of breast muscle cooked sous vide to an internal temperature of 59 °C, with the highest value obtained for Barnacle Goose (17.1%) and similar values (12.7–14.5%) in the other three species (GRE, BEA and Canada goose). Kokoszyński et al. (2022) and Uhlířová et al. (2018) reported that the genotype of farmed geese significantly affected the CL level in the breast meat, whereas Haraf et al. (2023), Lewko et al. (2017) and Gumułka and Połtowicz (2020) did not observe such an effect. Uhlířová et al. (2018) and Baeza et al. (2000) reported that cooking loss decreased as the age of waterfowl increased and suggested that the age- or genotype-dependent differences in CL may have been due to differences in protein solubility (especially for collagen) and fat content (Uhlířová et al., 2018).

Among the studied geese, White Kołuda breast muscles exhibited the highest fat content and also showed the highest expressible water and cooking loss. Previous studies have suggested that increased intramuscular fat deposition may be associated with a tendency toward lower water-holding capacity, which in turn may result greater meat weight losses during thermal processing (Daszkiewicz et al., 2005). In this context, the breeding system of geese is also important (Sari et al., 2015). In addition, the cooking method, temperature, time, presence of skin, pH and pre-cooking treatments have also been suggested as potential factors affecting the amount of cooking loss from muscle tissue (Wołoszyn et al., 2020).

The average cooking time of the breast muscles, irrespective of the species, breed, and type of geese, was 31.2 min (Table 4). The time is very close to the value of 30 min reported by Wołoszyn et al. (2020) for the breast muscles of WK geese, which may be due to the use of similar cooking parameters (average sample weight 370 g, water bath, endpoint temp. 75 °C).

### Colour of raw muscles

The colour attributes in the CIE system for wild geese were similar (*P* > 0.05) for both the breast muscle (average for L* = 28.7, a* = 9.6 and b* = 7.4) and leg muscle (L* = 33.6, a* = 12.0 and b* = 0.2) (Table 5). In contrast, significant (*P* < 0.05) breed differences (except for a* and b* in the legs) were noted in the group of domestic geese. In addition, the type of geese (wild vs. domestic) had a significant (*P* < 0.05) effect on the colour indices of the breast muscle, with higher values for L*, a* and b* observed for domestic geese (L* = 32.2, a* = 10.8 and b* = 8.6), due to the results obtained for the WK breed (Table 5). The leg muscles of wild geese, on the other hand, were significantly (*P* < 0.05) darker (L* = 33.6 vs. L* = 37.4) and redder (a* = 12.0 vs. a* = 9.4) than in domestic geese, with similar (*P* > 0.05) levels of yellowness (10.2 > b* < 10.0). The darker colour of wild goose meat observed in the present study may be associated with a higher concentration of heme pigments in their muscles. Myoglobin content is closely related to the metabolic characteristics of muscle fibres and is typically higher in muscles with greater oxidative capacity (Dobrzyńska et al., 2025; Geldenhuys et al., 2013). In wild, migratory geese, increased locomotor activity and sustained flight may promote the development of more oxidative muscle fibres, which could contribute to the darker colour compared with domestic birds (Geldenhuys et al., 2013; Saunders and Fedde, 1991). Söderquist et al. (2022) showed no significant differences in instrumental colour attributes for breast muscle between four species of geese (GRE vs. BEA vs. Barnacle Goose vs. Canada Goose; *P* > 0.066), and the ranges of values reported by the authors for L* (30.5–33.9), a* (9.3–12.2) and b* (6.3–8.6) are in close agreement with the results of the present study (Table 5). It is also worth noting that the colour attributes (L*, a* and b*) of the meat of the BI breed were more similar to the values obtained for wild geese than to the values for WK, which may be linked to both the origin of the birds (native breed vs. commercial hybrid) and their age (4 years vs. 17 weeks). A number of previous studies have confirmed the effect of the genotype and variety of geese on meat colour (Okruszek et al., 2008; Kowalczyk et al., 2013; Tůmová and Uhlířová, 2013; Lewko et al., 2017; Boz et al., 2019). Also, as birds age their meat becomes darker (Boz et al., 2017; Uhlířová et al., 2018; Weng et al., 2021; Wang et al., 2023) and less yellow (Boz et al., 2017; Uhlířová et al., 2018; Wang et al., 2023). In the case of the a* index, research results are inconclusive: some researchers indicate that it is higher in older geese (Weng et al., 2021), others report no significant changes (Boz et al., 2017; Uhlířová et al., 2018; Wang et al., 2023), and others have reported a decrease (Boz et al., 2017).

**Table 5:** Colour characteristics (CIE L*a*b*), total heme pigments and TBARS values of meat from three species of wild geese and two breeds of domestic geese (means; SEM).

Item	Goose type	Wild			**Wild** **Average**	SEM	Domestic		**Domestic** **Average**	SEM
	Muscle	Greater White-fronted (GWF)	Greylag (GRE)	Bean (BEA)			White Kołuda (WK)	Biłgorajska (BI)		
L*										
	Breast	†28.3	†29.2	†28.5	**†28.7x**	0.2	†34.2b	†29.5a	**†32.2y**	0.3
	Leg	†33.8	†33.3	†33.7	**†33.6x**	0.2	†39.6b	†35.3a	**†37.4y**	0.3
a*										
	Breast	†9.7	†9.2	†9.8	**†9.6x**	0.1	†12.0b	9.3a	**†10.8y**	0.1
	Leg	†12.0	†12.0	†12.1	**†12.0y**	0.1	†9.3	9.4	**†9.4x**	0.2
b*										
	Breast	†7.5	†7.3	†7.3	**†7.4x**	0.1	†10.6b	†6.0a	**†8.6y**	0.2
	Leg	†10.0	†10.1	†10.5	**†10.2**	0.1	†9.7	†10.2	**†10.0**	0.2
Total heme pigments, µg hematin/g										
	Breast	†231.3a	†308.8b	†281.6ab	**†275.4y**	10.9	†126.8	122.0	**†125.0x**	4.0
	Leg	†165.7ab	†188.9b	†160.4a	**†172.8y**	4.8	†84.0a	114.9b	**†97.3x**	4.7
TBARS, mg MDA/kg										
	Breast	†1.84	2.12	2.08	**†2.01y**	0.15	†1.35b	†0.27a	**0.95x**	0.11
	Leg	†2.68	2.61	2.63	**†2.64y**	0.15	†0.81	†0.69	**0.76x**	0.03

a, b - means with different letters in a row are significantly different at *P* < 0.05 (effect of goose species or breed - significance for three species of wild geese or two domestic breeds); x, y - means with different letters in a row are significantly different at *P* < 0.05 (effect of goose type – wild vs. domestic); † - means denoted with symbol in a column are significantly different at *P* < 0.05 (effect of muscle type – breast vs. leg); SEM – standard error of the mean.

Comparison of the colour attributes of the muscles revealed that the breast muscle of wild geese, irrespective of the species, was darker and less red and yellow than the leg muscle (Table 5). Geldenhuys et al. (2013) observed similar results in the wild Egyptian Goose, i.e. lower values for L* a* and b* in the breast muscle than in the thigh muscle (L* 28.61 vs. 32.57, a* 16.57 vs. 17.22, and b* 9.58 vs. 10.43). Similar differences between muscles in wild Egyptian Goose have been reported by Abd El Rahman et al. (2024). In the case of domestic breeds of geese, especially when slaughtered at a young age, it is notable that the breast muscle is darker than the leg muscle (as in wild geese), but it is usually also redder (Okruszek, 2012; Oz and Celik, 2015; Boz et al., 2019; Kokoszyński et al., 2022; Haraf et al., 2023). The higher L* value of the leg muscle, despite generally higher pH (which is usually accompanied by darker meat colour), is most often explained as the result of the muscle fibre composition and higher content of intramuscular fat, which increases the degree of light scattering and is generally lighter than muscle tissue (Geldenhuys et al., 2013).

### TBARS and total heme pigments

The meat of wild geese can have different quality traits to those of domesticated geese due to their natural diet, their level of activity, and the age they reach. The meat of wild animals is darker than that of farm animals, most likely due to their higher level of physical activity (Hoffman, 2000), which directly affects muscle fibre composition, with predominance of red type I and intermediate IIA fibres (Geldenhuys et al., 2013). The greater physical activity of wild geese, including flying (breast muscle), walking, swimming, and diving (leg muscles), also results in a higher myoglobin concentration in the muscles, mainly in order to increase their ability to bind and transport oxygen (Lawrie and Ledward, 2006; Geldenhuys et al., 2013). This is also reflected in the results of the present study, as wild geese had about twice the content of total heme pigments as domestic geese (275.4 µg/g vs. 125.0 µg/g in the breast and 172.8 µg/g vs. 97.3 µg/g in the leg; *P* < 0.05). The content of pigments was significantly (*P* < 0.05) the highest in GRE geese (308.8 µg/g in the breast and 188.9 µg/g in the leg), and the lowest in the breast of GWF (231.3 µg/g) and the leg of BEA (160.4 µg/g).

No significant differences were shown between species of wild geese for the lipid oxidation index (Table 5). However, the TBARS value was significantly (*P* < 0.05) higher in wild geese than in domestic geese (2.01 mg MDA/kg vs. 0.95 mg MDA/kg in the breast and 2.64 mg MDA/kg vs. 0.76 mg MDA/kg in the leg). The difference was most likely influenced by the twofold higher content of heme pigments in the meat of wild geese. Heme pigments (a source of strongly pro-oxidant iron) have been shown to play a key role in catalysing muscle lipid peroxidation (Min et al., 2008). Furthermore, the susceptibility of muscle to oxidation depends not only on the content of fat, but also on its composition. Muscles with a greater proportion of red fibres (which, as mentioned above, most likely applies to wild geese) are more sensitive, because they contain not only more iron, but also more phospholipids, rich in polyunsaturated fatty acids, which are highly susceptible to oxidative changes (Min et al., 2008; Faustman et al., 2010). Hence the higher TBARS values observed in the leg muscle compared to the breast muscle in the present study (2.64 mg MDA/kg vs. 2.01 mg MDA/kg; *P* < 0.05) may be a reflection of the relationships described above.

The literature lacks data on the degree of lipid oxidation in the meat of wild geese, while for domestic geese (WK breed), the TBARS value of fresh meat (< 72 h post mortem) ranges from 0.36 to 1.22 mg MDA/kg for breast muscle and from 0.44 to 1.15 mg MDA/kg for leg muscle (Karwowska et al., 2017; Wereńska et al., 2022). An increase in oxidation products has also been observed during frozen storage, reaching from 0.68 to 1.74 mg MDA/kg for breast muscle and from 1.06 to 1.31 mg MDA/kg for leg muscle after six months (Karwowska et al., 2017; Wereńska et al., 2022).

### Colour of cooked meat

The cooked breast muscle of GWF wild geese had significantly (*P* < 0.05) the highest lightness value and at the same time the lowest level of redness, compared to GRE and BEA; BEA also had significantly (*P* < 0.05) the lowest level of yellowness. Compared to domestic geese, the cooked breast of wild geese was darker (L* = 46.1 vs. 52.9; *P* < 0.05) and redder (a* = 12.1 vs. 10.3; *P* < 0.05), with similar levels of yellowness (b* = 14.8 vs. 14.4; *P* > 0.05). Measurement of the colour of cooked meat can provide reliable information on its qualitative traits and is just as important as in the case of raw meat, as the colour of cooked meat is an important factor influencing its acceptance by consumers (Wołoszyn et al., 2020; Kaliniak-Dziura et al., 2022). Colour analysis carried out by Wereńska (2023) and Wołoszyn et al. (2020) showed that irrespective of the cooking method (water bath, grilling, roasting, frying, sous-vide, microwave, or stewing), the breast muscles of WK geese became lighter (higher L* value) and more yellow (higher b* value), and at the same time less red (lower a* value) than raw meat. Similar colour changes caused by heat treatment of veal steaks from the longissimus thoracis muscle have been reported by Kaliniak-Dziura et al. (2022). The results of the present study for breast muscle of WK geese before and after cooking (Table 5, Table 6) are consistent with the findings of the authors cited. The colour of meat after cooking depends on numerous factors, among which the most important endogenous factors include the amount of heme pigments, redox status of myoglobin, pH, muscle type, intramuscular fat content, and antioxidant status. The most commonly mentioned exogenous factors include the system of packaging and storing meat, freezing-thawing conditions, and the type of heat treatment (King and Whyte, 2006). The increase in the L* value of meat after cooking is due to the increased reflection and scattering of light by denatured and aggregated sarcoplasmic and myofibrillar proteins (Wereńska, 2023). Lighter colour of cooked meat is desirable because it is more acceptable to consumers (Kaliniak-Dziura et al., 2022; Wereńska, 2023). A higher b* value is associated with accelerated formation of metmyoglobin from the other redox forms of myoglobin during aerobic heating, and its further denaturation leads to the formation of the dull brown pigment globin haemochromogen, also known as ferrihaemochrome (King and Whyte, 2006; Wołoszyn et al., 2020). Thermal denaturation of meat pigments, especially myoglobin, leads to a decrease in the a* value, with redness intensity inversely proportional to the degree of myoglobin denaturation. It should be noted, however, that the denaturation temperature of the various redox forms of myoglobin (deoxy-, oxy-and metmyoglobin) is not identical, and among them, deoxymyoglobin is the most heat-resistant. Higher pH protects myoglobin against thermal denaturation, allowing meat to retain its red or pink colour during and after cooking (King and Whyte, 2006). Geldenhuys et al. (2014) showed that heat-treated breast muscle of Egyptian Goose was darker, more red, and less yellow than five other kinds of meat (breast muscle of wild guineafowl vs. Pekin duck vs. broiler chicken vs. ostrich fan fillet vs. ostrich moon steak). According to the authors, this is explained by how the geese were obtained during hunting. The birds were shot in flight, causing them to experience stress before they died, and furthermore their higher level of physical activity translated into a higher proportion of red fibres in the meat. Both factors contribute to higher meat pH. In effect, a positive correlation was shown between pH and redness (*r* = 0.434; *P* = 0.008) and a negative correlation between pH and lightness (*r* = −0.534; *P* = 0.001) (Geldenhuys et al., 2014). These relationships may therefore explain the observations in the present study following heat treatment, i.e. darker meat from wild geese than from domestic breeds, as well as the increase in redness, in contrast to the commercial WK breed.

**Table 6:** Colour characteristics (CIE L*a*b*) of cooked meat and texture characteristics of raw and cooked breast meat from wild geese and domestic goose breeds (means; SEM).

Item	Goose type	Wild			**Wild Average**	SEM	Domestic		**Domestic Average**	SEM
		Greater White-fronted (GWF)	Greylag (GRE)	Bean (BEA)			White Kołuda (WK)	Biłgorajska (BI)		
*Colour characteristics of cooked breast*										
L*		48.4b	44.8a	43.6a	**46.1x**	0.3	53.2	52.6	**52.9y**	0.3
a*		11.3a	12.8b	12.8b	**12.1y**	0.1	9.7a	11.0b	**10.3x**	0.2
b*		15.4b	15.0b	13.4a	**14.8**	0.1	14.0	14.9	**14.4**	0.2
*Texture traits of raw breast*^1^										
Shear force, N		26.6	26.7	31.0	**27.7x**	1.1	36.2a	63.9b	**50.4y**	2.9
Shear energy, *N* × mm		165.7	172.0	193.7	**174.8x**	7.2	190.4a	357.3b	**276.2y**	17.6
*Texture traits of cooked breast*^1^										
Shear force, N		40.3a	51.6b	39.7a	**44.0x**	1.1	51.6a	78.2b	**65.4y**	2.4
Shear energy, *N* × mm		152.6a	192.9b	134.4a	**162.1x**	4.7	178.4a	268.2b	**225.1y**	10.8
MORS shear force, N		12.6	13.0	13.6	**13.0**	0.2	15.0b	12.2a	**13.7**	0.3
MORS shear energy, *N* × mm		119.3	131.0	131.2	**126.1**	2.1	151.4b	106.2a	**130.9**	3.5

a, b - means with different letters in a row are significantly different at *P* < 0.05 (effect of goose species or breed - significance for three species of wild geese or two domestic breeds); x, y - means with different letters in a row are significantly different at *P* < 0.05 (effect of goose type – wild vs. domestic); SEM – standard error of the mean.

1 Shear force and shear energy determined using the ‘V’ slot blade test; MORS, Meullenet-Owens Razor Shear Blade test.

### Instrumental texture measurements

Texture analysis by the V-blade test showed that the shear force (SF) and shear energy of the raw breast muscle were comparable in all species of wild geese (on average 27.7 N and 174.8 *N* × mm), while after heat treatment they were significantly (*P* < 0.05) the highest in GRE (51.6 N and 192.9 *N* × mm), compared to GWF and BEA (39.7–40.3 N and 134.4–152.6 *N* × mm) (Table 6). Significantly (*P* < 0.05) higher values for the texture parameters of the breast muscle in the V-blade test, both before and after cooking, were obtained for domestic geese (raw 50.4 N and 276.2 *N* × mm and cooked 65.4 N and 225.1 *N* × mm) than for wild geese, with the highest numerical values obtained for the BI breed among all species and breeds tested (Table 6). The results of the other test, performed using the Meullenet-Owens Razor Shear Blade (MORS), showed no significant differences between species of wild geese or the type of geese (wild vs. domestic; *P* > 0.05), with cooked breast of the WK breed showing the highest (numerical) values for both parameters in this test.

Shear force is a widely used instrumental measure of meat tenderness, with lower values indicating more tender meat. The measurement is usually made on cooked meat, since meat – including goose meat – is rarely eaten raw. Nevertheless, evaluation of the SF of raw meat and the range of changes induced in it by heat treatment may be interesting. Gronowicz et al. (2018) compared the shear force of raw breast muscles from four native breeds of geese (Lubelska, Kielecka, Pomorska, and Podkarpacka) and WK commercial hybrids slaughtered at the age of 17 weeks and showed no significant differences between breeds, with values ranging from 28.89 N to 36.74 N. Higher SF was observed after cooking, with very similar values for all genotypes (50.45–52.60 N). Similarly, SF ranges given by other authors for cooked breast from WK commercial hybrids (49.72–72.82 N; Kapkowska et al., 2011; Gumułka and Połtowicz, 2020; Wołoszyn et al., 2020), Eskildsen Schwer Goose (39.22–42.05 N; Uhlířová et al., 2018), Polish and Czech native breeds of geese (38.92–50.22 N; Kapkowska et al., 2011; Uhlířová et al., 2018; Gumułka and Połtowicz, 2020), and wild Egyptian Goose (46.29–61.08 N; (Geldenhuys et al., 2014, 2016a) are comparable to the results of the present study (Table 6).

The meat of older geese (13–43 weeks old) has higher SF than that of younger geese (8–10 weeks old; Uhlířová et al., 2018; Weng et al., 2021; Wang et al., 2023), most likely due to the increasing diameter of muscle fibres with age (Uhlířová et al., 2018; Weng et al., 2021) and to the content of collagen, including its insoluble fraction (Geldenhuys et al., 2016b). Kokoszyński et al. (2022) showed no significant differences in the SF of the breast muscle of four-year-old geese of the breeds Landes, Slovak White, and Kuban; the values in the range of 93.9–129.7 N were somewhat higher than in the present study for four-year-old BI geese (Table 6). However, the increase in SF with age observed in domestic goose breeds does not seem to apply to species of wild geese. All wild geese in the present study were older than 18 months. The SF values of cooked breast muscle from GWF and BEA geese were numerically lower, while the values for GRE were comparable to those for 17-week-old WK geese, and at the same time lower than in four-year-old BI geese (Table 6). As mentioned above, wild geese differ from domestic geese in their diet and level of physical activity as well as microstructural characteristics and muscle fibre type composition, which to some extent may modify meat texture. As demonstrated earlier, muscles with a predominance of red fibres have a smaller diameter, which is beneficial for their texture (Buzała et al., 2014). Therefore, there is a need for further studies to provide a detailed assessment of the traits of muscle fibres of different species of wild geese.

The Meullenet-Owens Razor Shear test (MORS) is an indirect (instrumental) method for assessing the tenderness of poultry meat, and its reliability is similar to that of the most common method, the shear force test. In contrast to SF, MORS requires less sample preparation, because an entire intact fillet can be sheared (without cutting out samples) (Lee et al., 2008). Goose meat is very rarely evaluated using this test. Dobrzyńska et al. (2025), in cooked breast muscle of 17-week-old crosses of the Tapphorn and Eskildsen meat lines, obtained higher MORS force and energy than in the present study, ranging from 15.27 to 17.14 N and from 211.01 to 245.71 *N* × mm, respectively. The MORS test is most commonly used to assess the tenderness of broiler breast fillets, and the ranges of force and energy values are usually 8.9–14.0 N and 108.2–192.9 *N* × mm (Lee et al., 2008; Mueller et al., 2023).

### Sensory evaluation

In the group of wild geese, BEA meat had significantly (*P* < 0.05) the highest overall quality and aroma intensity (8.5 and 5.9 pts, respectively), while GWF and GRE received the same lower scores (7.9 and 4.2 pts, respectively) (Table 7). Meat flavour in the wild species was rated very high (> 7.5 pts), with BEA breast muscle receiving significantly (*P* < 0.05) the highest score and GWF the lowest. The juiciness and tenderness of the meat were rated equally high (> 8.0 pts), and together with off-odours (on average 2.4 pts) did not differ significantly (*P* > 0.05) between species. Söderquist et al. (2022) reported similar results for sensory evaluation of the breast muscle of four species of wild geese (Barnacle, BEA, GRE and Canada Goose). They showed that the meat of BEA and GRE geese had significantly more intense overall meat and iron odour than the meat of Canada geese. The meat of the Barnacle Goose was more tender (*P* < 0.001) than the meat of the other species. No significant differences between species were shown for flavour or juiciness (Söderquist et al., 2022).

**Table 7:** Sensory evaluation of cooked breast muscles of wild geese and domestic goose breeds, rated on a 10-point scale (means; SEM).

Trait	Goose type	Wild			**Average**	SEM	Domestic	SEM
		Greater White-fronted (GWF)	Greylag (GRE)	Bean (BEA)			White Kołuda (WK)	
Meat aroma		4.2a	4.2a	5.9b	**4.8x**	0.2	7.2y	0.3
Off-odours		3.0	2.1	2.2	**2.4y**	0.2	0.4x	0.1
Meat flavour		7.7a	8.4ab	8.7b	**8.3**	0.1	8.5	0.1
Off-flavours		2.7b	1.9ab	1.0a	**1.9y**	0.2	0.5x	0.1
Juiciness		8.5	8.4	8.1	**8.3y**	0.1	7.6x	0.2
Tenderness		8.7	8.7	8.8	**8.7y**	0.1	7.8x	0.2
Overall quality		7.9a	7.9a	8.5b	**8.1**	0.1	8.1	0.1

a, b - means with different letters in a row are significantly different at *P* < 0.05 (effect of goose species); x, y - means with different letters in a row are significantly different at *P* < 0.05 (effect of goose type – wild vs. domestic White Kołuda®); SEM – standard error of the mean.

Compared to the wild species, the meat of domestic WK geese had significantly (*P* < 0.05) the most distinct meat aroma (4.8 vs. 7.2 pts), while the scores for meat flavour (8.3 vs. 8.5 pts) and overall quality were similar (8.1 pts for both types; *P* > 0.05). The meat of wild geese had significantly (*P* < 0.05) higher intensity of off-flavours (2.4 vs. 0.4 pts) and off-odours (1.9 vs. 0.5 pts) than WK; the negative notes most frequently indicated by the panellists were a livery and earthy odour, and in the case of flavours, livery, metallic, and bitter (data not shown). Söderquist et al. (2022) also found relatively high intensity of iron odour in the meat of wild geese (about 40 pts on a 100-pt scale), as well as of liver (about 45 pts), metallic (about 50 pts) and grass (about 35 pts) flavours. Geldenhuys et al. (2014) stress that the breast muscle of the wild Egyptian Goose has a specific sensory profile including a strong odour and gamey flavour, together with a distinct metallic/livery aftertaste, which clearly distinguishes it from the meat of other bird species (wild guineafowl, domestic Pekin duck, broiler chicken, and ostrich). This is linked to high content of iron and polyunsaturated fatty acids (Geldenhuys et al., 2014). In addition, Egyptian Goose meat was the least tender, and also less juicy than wild guineafowl and broiler chicken, which may be due to high physical activity of the breast muscles associated with flight (Geldenhuys et al., 2014). Contrasting results were observed in the present study. Although goose meat was not compared to other poultry species, it should be noted that the breast muscle of wild geese had significantly (*P* < 0.05) more favourable texture parameters than that of WK geese for juiciness (8.3 vs. 7.6 pts) and for tenderness (8.7 vs. 7.8 pts) (Table 7). These differences, in the present study and in cited research (Geldenhuys et al., 2014), may be due to natural interspecific differences in the birds, as well as to dietary factors. Geldenhuys et al. (2016a) showed that the meat of the wild Egyptian Goose on a diet rich in n-3 polyunsaturated fatty acids (including aquatic plants, aquatic invertebrates, and other aquatic and terrestrial green plants) has more distinct gamey, metallic, and fishy notes, whereas the meat of birds fed a grain-based diet has a milder sensory profile, associated with sweet-oily-duck and beef odours and flavours.

## Conclusions

The study clearly demonstrated significant differences in carcass traits and meat quality among species of wild geese and between types of birds (wild vs. domestic). Among the wild goose species, Greylag exhibited the most favourable slaughter characteristics, with the highest body weight and the greatest mass of edible carcass components, especially the breast and leg muscles, whereas the Greater White-fronted Goose showed the lowest values. In comparison with wild birds, White Kołuda® (a domestic goose breed), were characterized by substantially higher body weight and heavier edible parts, although the percentage of breast muscle was markedly higher in wild geese.

Wild goose meat was characterized by high protein content and relatively low (in breast) or moderate (in leg) intramuscular fat, as well as darker colour, higher heme pigment concentration, and greater susceptibility to lipid oxidation. Domestic geese, on the other hand, contained more intramuscular fat and water and exhibited lower pH and higher torrymeter values. Despite these physicochemical differences, sensory evaluation performed for wild geese and the White Kołuda® breed indicated high overall acceptability of the breast muscle, with wild goose meat perceived as more juicy and tender, whereas White Kołuda® meat showed a more intense meat aroma.

Overall, the observed differences most likely reflect the morphological and physiological consequences of domestication, including reduced locomotor activity and selection for rapid growth and meat production. The findings also fill an important gap in the literature on the slaughter traits and the composition and quality of meat from three different species of wild geese, confirming that this meat represents a nutritionally valuable and sensory-acceptable product that may constitute an alternative source of poultry meat and a potential resource within sustainable wildlife management systems.

Importantly, the present study provides one of the most comprehensive comparative datasets describing carcass traits and meat quality characteristics of wild European geese in relation to selected domestic goose breeds. However, it also has certain limitations due to restricted availability of biological material from the Biłgorajska breed, and analyses were therefore limited to selected physicochemical characteristics of the meat. Further research is warranted, including analyses of muscle fibre characteristics, lipid and proteomic profiles, to better understand the mechanisms and interspecific differences underlying the meat quality of wild geese.

## CRediT authorship contribution statement

**Paweł Piekarczyk:** Writing – review & editing, Writing – original draft, Visualization, Resources, Methodology, Investigation, Formal analysis, Data curation, Conceptualization. **Piotr Domaradzki:** Writing – review & editing, Writing – original draft, Validation, Supervision, Project administration, Methodology, Investigation, Funding acquisition, Data curation, Conceptualization. **Mariusz Florek:** Writing – review & editing, Validation, Formal analysis, Conceptualization. **Marek Kowalczyk:** Investigation, Methodology, Writing – review & editing. **Barbara Topyła:** Resources, Investigation. **Katarzyna Tajchman:** Resources, Methodology, Investigation.

## Disclosures

The authors declare that they have no known competing financial interests or personal relationships that could have appeared to influence the work reported in this paper.

## Appendix Supplementary materials

Figure S1. Experimental design and analysis timeline Image, application 1

## Footnotes

Supplementary material associated with this article can be found, in the online version, at doi:10.1016/j.psj.2026.106877. Figure S1. Experimental design and analysis timeline
