# Supplementary figures and images for: Carcass characteristics and physicochemical and sensory properties of meat from three species of wild geese hunted in Poland
Source: Poult Sci. 2026 Mar 28;105(7):106877. doi: 10.1016/j.psj.2026.106877 (PMC13090970; doi:10.1016/j.psj.2026.106877)

**Supplementary materials**


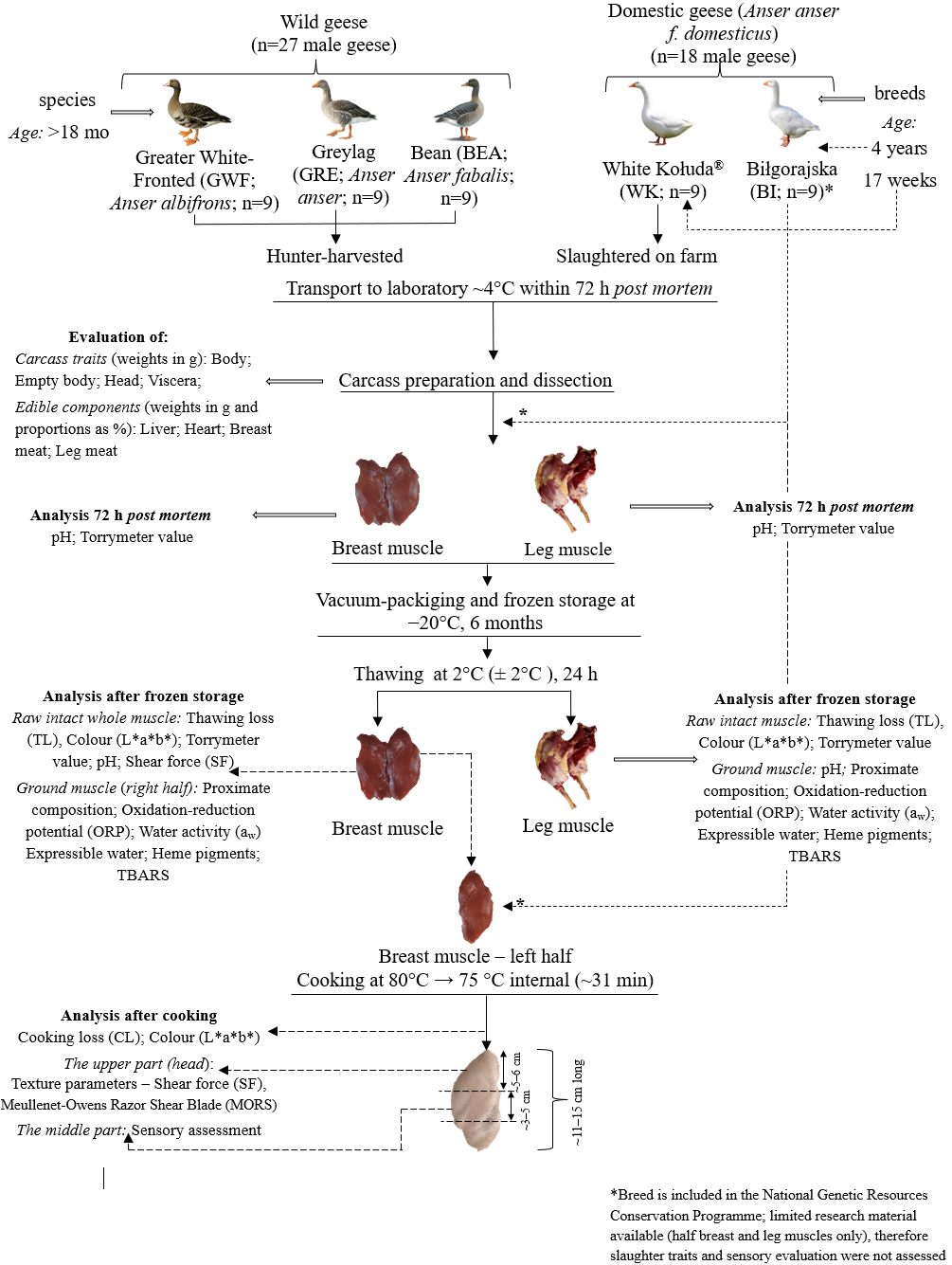


Figure S1. Experimental design and analysis timeline

Supplement: Supplementary file 1 — Figure S1. Experimental design and analysis timeline [file mmc1.docx]
